# Efficacy and safety of levosimendan in patients with sepsis: a systematic review and network meta-analysis

**DOI:** 10.3389/fphar.2024.1358735

**Published:** 2024-03-08

**Authors:** Ruimin Tan, He Guo, Zinan Yang, Huihui Yang, Qinghao Li, Qiong Zhu, Quansheng Du

**Affiliations:** ^1^ School of Clinical Medical, North China University of Science and Technology, Tangshan, Hebei, China; ^2^ Critical Care Department, Hebei General Hospital, Shijiazhuang, Hebei, China; ^3^ School of Graduate, Hebei Medical University, Shijiazhuang, Hebei, China; ^4^ Department of Orthopaedics, The People’s Hospital of Shizhu, Chongqing, China

**Keywords:** levosimendan, sepsis, systematic review, network meta-analysis, intensive care unit

## Abstract

**Objective:** We conducted a systematic review to assess the advantages and disadvantages of levosimendan in patients with sepsis compared with placebo, milrinone, and dobutamine and to explore the clinical efficacy of different concentrations of levosimendan.

**Methods:** PubMed, Web of Science, Cochrane Library, Embase, CNKI, Wanfang data, VIP, and CBM databases were searched using such keywords as simendan, levosimendan, and sepsis. The search time was from the establishment of the database to July 2023. Two researchers were responsible for literature screening and data collection respectively. After the risk of bias in the included studies was evaluated, network meta-analysis was performed using R software gemtc and rjags package.

**Results:** Thirty-two randomized controlled trials (RCTs) were included in the network meta-analysis. Meta-analysis results showed that while levosimendan significantly improved CI levels at either 0.1 µg/kg/min (mean difference [MD] [95%CrI] = 0.41 [−0.43, 1.4]) or 0.2 µg/kg/min (MD [95%CrI] =0.54 [0.12, 0.99]). Levosimendan, at either 0.075 µg/kg/min (MD [95% CrI] =0.033 [−0.75, 0.82]) or 0.2 µg/kg/min (MD [95% CrI] = −0.014 [−0.26, 0.23]), had no significant advantage in improving Lac levels. Levosimendan, at either 0.1 µg/kg/min (RR [95% CrI] = 0.99 [0.73, 1.3]) or 0.2 µg/kg/min (RR [95% CrI] = 1.0 [0.88, 1.2]), did not have a significant advantage in reducing mortality.

**Conclusion:** The existing evidence suggests that levosimendan can significantly improve CI and lactate levels in patients with sepsis, and levosimendan at 0.1 µg/kg/min might be the optimal dose. Unfortunately, all interventions in this study failed to reduce the 28-day mortality.

**Systematic Review Registration:** https://www.crd.york.ac.uk/prospero/display_record.php?ID=CRD42023441220.

## 1 Introduction

Sepsis is one of the leading causes of admission to the intensive care unit (ICU), accounting for approximately 20%–30% of ICU admissions (Vincent et al., 2014). About 15% of sepsis patients progress to septic shock (Fleischmann et al., 2016; Liu, 2017). Up to 18%–40% of septic shock patients were complicated with heart failure and myocardial depression (Liu, 2017; Ravikumar et al., 2021; Koya and Paul, 2023). If they are not treated in time, the mortality rate can reach 70%–90% (Dugar et al., 2020). The occurrence of these complications may be related to the imbalance of myocardial energy metabolism, the production of a large number of negative regulatory factors in myocardium, and apoptosis of myocardial cells (Zuo et al., 2023). Therefore, in the treatment, attention should be paid not only to the control of sepsis infection, but also to the protection of cardiac function (Vincent and Bakker, 2021; Chen and Fu, 2023).

Levosimendan is a Ca^2+^ sensitizer that enhances myocardial contractility by increasing the sensitivity of cardiac troponin to Ca^2+^, but does not increase myocardial oxygen consumption and cause calcium overload, thus avoiding arrhythmias caused by traditional positive inotropic drugs (Sun et al., 2023). Because of its good cardiotonic effect, it has been widely used in the treatment of acute heart failure and cardiogenic shock (Fan et al., 2019). The results of current studies are inconsistent as to whether levosimendan is effective in the treatment and overall prognosis of sepsis patients. Studies by Morelli et al. showed that levosimendan can increase cardiac output, reduce lactate levels, improve intestinal microcirculation, and modulate inflammatory responses (Matejovic et al., 2005; Morelli et al., 2005; Morelli et al., 2006; Tasouli et al., 2007; Pan et al., 2019; Yang et al., 2019). However, other studies indicated that levosimendan does not improve organ dysfunction or reduce mortality in patients with sepsis (Antcliffe et al., 2019). Hence, further investigation is needed to determine the therapeutic value of levosimendan in sepsis (Saevik et al., 2021).

Given the large heterogeneity of control groups in existing studies on levosimendan and the different regimens of levosimendan used in different studies, we want to explore the advantages and disadvantages of levosimendan compared with placebo, milrinone, and dobutamine in patients with sepsis and the clinical efficacy of different concentrations of levosimendan. A network meta-analysis can analyze more than two interventions simultaneously, based on indirect comparisons or a combination of indirect and direct comparisons. It allows for quantitative comparisons of different interventions for the same disease and ranks them according to the effect on a certain outcome measure. This study used systematic review and network meta-analysis to synthesize the available evidence. The objective of this study is to directly and indirectly compare the efficacy of dobutamine, milrinone, and levosimendan in patients with sepsis and explore the efficacy of levosimendan at different doses.

## 2 Methods

### 2.1 Protocol and registration

This study followed the PRISMA guideline (Hutton et al., 2015) and was pre-registered on the PROSPERO platform (registration number #CRD42023441220) (Tan et al., 2023).

### 2.2 Search strategy

The main terms used to construct the search strategy were simendan, levosimendan, and sepsis. PubMed, Web of Science, Cochrane Library, Embase, CNKI, Wanfang Data, VIP, and CBM were searched from the date of database inception to July 2023. In addition, the references of the included studies were traced to supplement relevant studies.

### 2.3 Literature screening

Literature screening, data extraction, and cross-checking were conducted by two independent researchers. In case of any disagreements, a third researcher was consulted for resolution. Reasons for excluding literature were clearly recorded, and an exclusion criteria list was created. Efforts were made to contact the original authors via email or phone for any missing data. During the screening and evaluation of literature, the titles and abstracts were initially reviewed to remove duplicates and articles that did not meet the requirements. Subsequently, the full texts were further examined to determine the included studies.

### 2.4 Eligibility criteria

#### 2.4.1 Inclusion criteria

1) Type of study: randomized controlled trials (RCT); 2) Participants: patients met the definition criteria of sepsis or septic shock in international guidelines at that time, age>18 years old; gender, nationality, race, source of infection, pathogen and course of disease were not limited; 3) Intervention measures and comparison: The control group was treated with dobutamine, milrinone or placebo, while the experimental group was treated with levosimendan intravenous injection or intravenous pump, and other conventional treatments were the same as those in the control group; 4) Outcome measures: Cardiac index (CI) was used as the primary outcome measure, while lactic acid (Lac) level and 28-day mortality were used as secondary outcome measures.

#### 2.4.2 Exclusion criteria

1) Studies that included underage patients; 2) duplicate publications; 3) studies without outcome measure data; 4) studies with incomplete data or unavailable data; 5) non-Chinese/English studies; and 6) animal experiments.

### 2.5 Bias risk assessment for included studies

The risk of bias was assessed using the RCT bias risk assessment tool built into Cochrane Review Manager (5.4.1). Two investigators independently evaluated the bias risk of the included studies, and cross-checked the evaluation results. Disagreements were discussed or adjudicated by a third investigator. The evaluation domains included: (1) whether the random allocation method was correct; (2) whether the allocation hiding method was used; (3) whether the blinding method was used; (4) whether the outcome data were complete; (5) whether there was selective reporting of study results; (6) other sources of bias. Each evaluation domain was judged as high, uncertain, or low risk of bias.

### 2.6 Data extraction

The following data were extracted: (1) general information, including publication country, publication year, publication journal, first author and title; (2) basic characteristics of study subjects, including sample size, gender composition, age distribution and disease severity; (3) study characteristics, including study objective, study type, administration method, dose, duration and results of levosimendan; (4) key elements of bias risk evaluation; (5) outcome indicators, including 28-day mortality, CI, and Lac.

### 2.7 Statistical analysis

The statistical analysis in this study was performed using the gemtc package (version 1.0-1) in R software (version 4.0.4). Mean difference (MD) was used for continuous data, odds ratio (OR) was used for binary data, and 95% confidence intervals (95%CrI) were obtained for each effect size (ES). The *I* ^*2*^ test was used to assess the heterogeneity among studies. A 95% CrI that does not include 0 (for continuous data) or 1 (for binary data) indicates a statistically significant difference. First, the “mtc.network” function was used to construct a network, followed by generating models using the “mtc.model”. Finally, Bayesian analysis was performed using the “mtc.run” function. In the “mtc.model” function, the likelihood/link was set to “binom/log” to compute the log risk ratio (logRR) with a 95% CrI based on collected binary data. The JAGS software (through the “rjags” package) was used for model estimation. Markov Chain Monte Carlo (MCMC) method was used to compute a fixed-effect model based on 5,000 adaptive simulations and 20,000 iterations. A ranking table was generated to display the relationships between all treatment concentrations, and the “exp” function was used to calculate the relative risk (RR) from the log risk ratio (LogRR). Additionally, a forest plot of relative effects was provided to visualize the relative effects of different treatment processes compared to the standard treatment process. The surface under the cumulative ranking curve (SUCRA) was calculated to compare the differences in treatment efficacy among different interventions. The convergence of iterations was quantified by calculating potential scale reduction factor (PSRF) values. The consistency assumption was tested using the node-splitting method, with inconsistency considered significant at *p* < 0.05. Furthermore, the “mtc.anohe” function was employed to test the homogeneity hypothesis. *I* ^*2*^ > 50% indicated significant heterogeneity. A sensitivity analysis was conducted by comparing the pooled results of the random-effects model with the fixed-effect model.

## 3 Results

### 3.1 Search results

**FIGURE 1:**
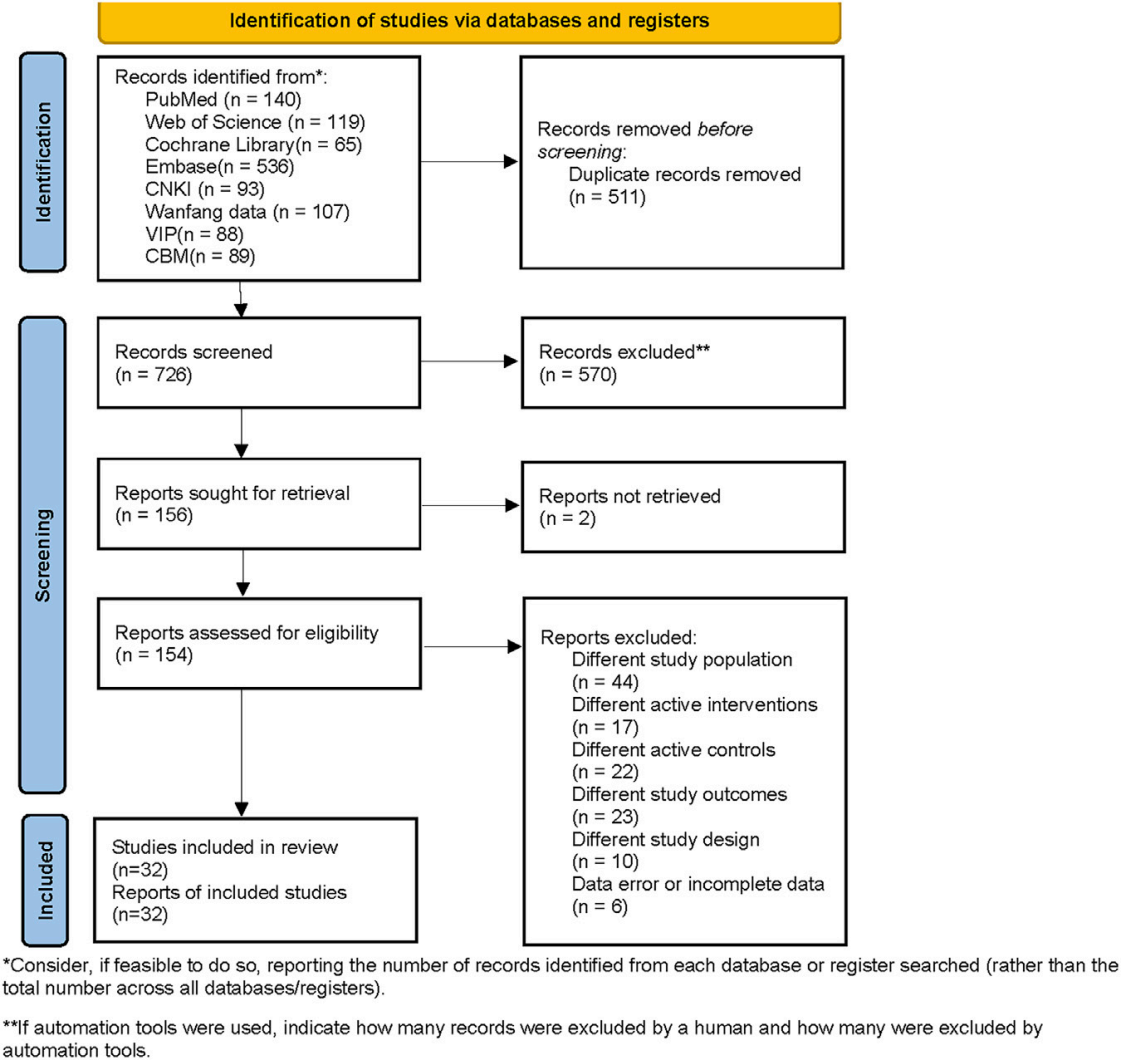
PRISMA 2020 flow diagram.

A total of 1,237 studies were retrieved, including 140 from PubMed, 119 from Web of Science, 65 from Cochrane Library, 536 from Embase, 93 from CNKI, 107 from Wanfang data, 88 from VIP, and 89 from CBM. The search strategies and results are listed in Supplementary Material S2. After filtering for duplicates and excluding studies that did not match PICOS based on titles and abstracts, a total of 38 full-text articles were retrieved. After carefully reading the entire texts, 32 studies (Morelli et al., 2005; Morelli et al., 2006; Morelli et al., 2010; Fang and Dong, 2014; Torraco et al., 2014; Huang et al., 2015; Gordon et al., 2016; Lai et al., 2016; Meng et al., 2016; Wu and Chang, 2016; Yan et al., 2016; Hajjej et al., 2017; Huang et al., 2017; Janssens, 2017; Wang D. et al., 2018; Wang W. et al., 2018; Lan et al., 2018; Su et al., 2018; Xu et al., 2018; Fan et al., 2019; Pan, 2019; Yang and Li, 2019; Bian et al., 2020; Liu et al., 2020; Lu et al., 2020; Shi and Lin, 2020; Liu et al., 2021; Yang and Yi, 2021; Hua et al., 2022; Chen and Fu, 2023; Sun et al., 2023; Zhou, 2023) (involving 2,813 patients) were included in the Bayesian analysis. The PRISMA flowchart is shown in Figure 1. Among them, there were 20 Chinese studies and 12 English studies.

### 3.2 Study characteristics

A total of 32 studies (involving 2,813 sepsis patients) were included, with 23 studies comparing two or more different inotropic drugs and 9 studies comparing single levosimendan or its combination with other drugs versus placebo. Four trials were multicenter and mainly conducted in developed regions such as Europe and North America. There were no significant differences in gender distribution and age distribution among the included studies, with most patients being middle-aged or elderly. In the assessment of initial disease severity, SAPS II scores were provided in four studies, APACHE II scores in 20 studies, and SOFA scores in 15 studies, indicating a generally consistent severity of the patients’ diseases. In the evaluation of initial cardiac function, 18 studies provided BNP levels and 19 studies provided CI levels, with no significant differences observed in the initial cardiac function of the patients (Table 1). Through the comparative analysis of study design, outcome measures, patient characteristics, and inclusion/exclusion criteria, we found that conducting a network meta-analysis was appropriate for the comprehensive evaluation of the evidence. Homogeneity and consistency assumptions were statistically confirmed (Supplementary Material S1).

**TABLE 1:** Characteristics of included studies.

Study	Region	sampleSize	Age (Mean,SD)	Gender	Severity	Cardiac function (pg/mL)	Microcirculation (mmol/L)
H, Huang 2015	China	51	66.03,4.07	21,30	APACHE Ⅱ 24.10,6.78	BNP 3798.14,220.78;CI 2.1,0.72	Lac 7.70,3.81
Z.Z.Lai 2016	China	38	52.5,16.11	14,24	SOFA 4.25,2.21;APACHE Ⅱ 19,4.07	BNP 437,93.46;CI 2.95,0.30	Lac 4.9,1.15
Y.P.Lan 2018	China	45	71.80,15.77	15,30	SOFA 16.78,5.25;APACHE Ⅱ 22.22,6.27	BNP 4504.51,1572.87;CI 2.35,0.36	Lac 12.20,3.30
Z.W.Lu 2020	China	40	69.5,7.00	17,23	SOFA 4.2,1.74;APACHE Ⅱ 22.5,8.42	BNP 533.45,109.45;CI 2.75,0.35	Lac 6.35,1.05
J.S.Shi 2020	China	90	59,5.07	41,49	NA	BNP 516.5,55.75;CI 2.2,0.70	NA
W.Wang 2018	China	84	65.61,10.79	28,56	APACHE Ⅱ 11.31,3.21	CI 2.21,0.61	Lac 8.83,2,91
Z.J.Yan 2016	China	60	73.4,13.30	15,45	APACHE Ⅱ 26.06,7.64	BNP 742.45,138.63;CI 2.05,0.47	NA
S.B.Yang 2019	China	84	62.05,0.59	34,50	NA	CI 2.07,0.63	NA
X.M.Zhou 2023	China	105	58.99,5.87	24,28	NA	CI 1.92,0.26	NA
C.G.Chen 2023	China	80	59.49,6.03	37,43	NA	BNP 206.87,15.98	Lac 3.33,1.05
X.S.Hua 2022	China	72	79.04, 8.92	30,42	SOFA 26.61, 7.77	BNP 2359.65,852.55	Lac 7.63,2.31
H.J.Liu 2020	China	120	62.61,6.99	42,78	SOFA 18.86,1.66	BNP 3700,870	Lac 10.75,1.43
H.J.Liu 2021	China	60	58.85,5.65	35,25	SOFA 12.50,1.69;APACHE Ⅱ 21.45,6.28	BNP 439.97,91.49	Lac 4.76,1.72
C.K.Pan 2019	China	72	65.90,6.21	33,39	SOFA 16.93,2.81;APACHE Ⅱ 29.81,5.08	NA	Lac 8.62,2.47
G.Q.Wu 2016	China	94	46.06,6.29	45,49	APACHE Ⅱ 20.05,4.96	BNP 596.41,53.44;CI 2.54,0.69	Lac 11.96,3.98
X.H.Bian 2020	China	32	41.35,9.82	19,13	SOFA 17.7,1.94;APACHE Ⅱ 20.9,1.40	NA	Lac 5.35,0.71
D.B.Wang 2018	China	48	76.4,7.13	15,33	SOFA 14.75,2.82;APACHE Ⅱ 21.95,2.85	BNP 2199.67,1637.85	Lac 3.5,2.87
Y.G.Huang 2017	China	63	63.10,6.66	21,42	SOFA 18.90,1.50;APACHE Ⅱ 21.85,3.68	BNP 420.19,52.69	NA
B.J.Su 2018	China	55	60.84,7.44	19,36	APACHE Ⅱ 25.11,3.02	BNP 526.98,29.86	NA
L.Yang 2021	China	80	62.1,6.6	40,42	SOFA 19,1.60;APACHE Ⅱ 24.09,5.35	BNP 1582.01,654.25	NA
C.X.Xu 2018	China	30	88,7.55	14,16	APACHE Ⅱ 23.6,5.73	BNP 5015.61,6365.50;CI 4.47,1.87	Lac 2.54,1.67
M.X.Fang 2014	China	36	61.55,7.10	9,27	SOFA 18,1.60;APACHE Ⅱ 23.8,2.24	NA	NA
Z.Fan 2019	China	126	62.70,6.19	63,63	SOFA 18.41,2.07	BNP 423.67,54.20;CI 2.27,0.55	Lac 10.39,3.25
A.C.Gordon 2016	the United Kingdom	515	67.30,13.44	226,289	SOFA 9.83,3.38;APACHE Ⅱ 25.53,7.10	CI 3.02,1.27	Lac 2.48,1.68
Z.Hajjej 2017	Italy	20	49.9,30.76	3,17	SAPS Ⅱ 56.22,18.49	CI 3.79,1.19	Lac 1.99,0.84
J.B.Meng 2016	China	38	52.8,15.68	14,24	SOFA 4.25,2.21;APACHE Ⅱ 18.95,4.38	CI 2.95,0.26	Lac 4.9,1.15
A.Morelli 2005	Italy	28	61.92,7.02	7,21	APACHE Ⅱ 24.08,1.85	CI 4.15,0.25	Lac 5.04,1.14
A.Morelli 2006	Italy	35	66.64,7.55	8,27	SAPS Ⅱ 50.03,10.42	CI 3.99,1.01	Lac 3.58,1.93
T.Sun1 2023	China	30	47.53,16.02	16,14	APACHE Ⅱ 18.97,4.16	BNP 3184,1509.82;CI 1.66,0.33	Lac 9.16,1.95
A.Morelli 2010	Italy	40	65.74,17.05	13,27	SAPS Ⅱ 54.56,13.45	CI 3.84,1.55	Lac 2.1,1.26
U.Janssens 2017	Germany	516	NA	227289	NA	NA	NA
A.Torraco 2014	Italy	26	68.64,17.91	7,19	SAPS Ⅱ 61.55,11.06	NA	NA

### 3.3 Quality evaluation

**FIGURE 2:**
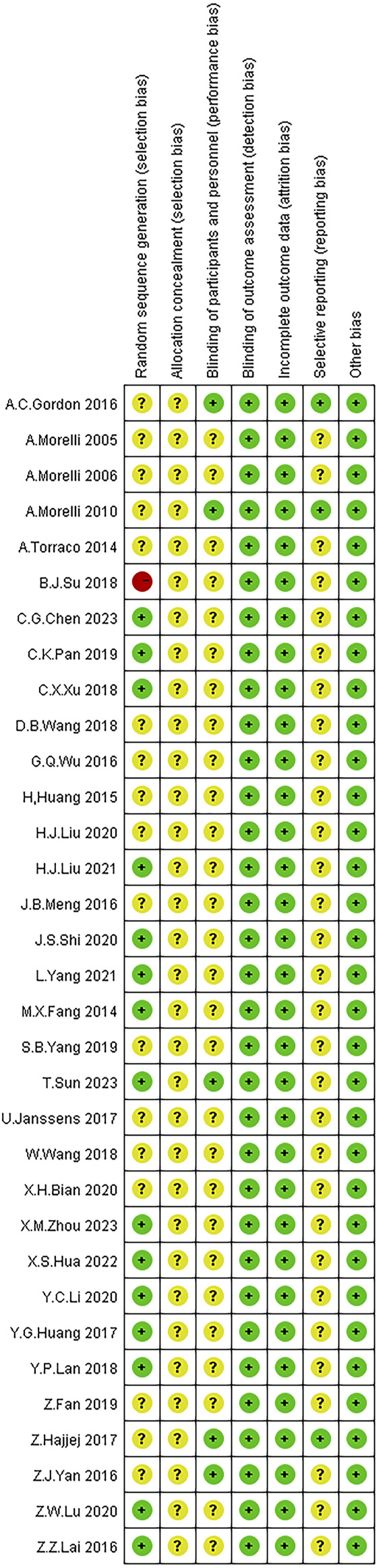
Risk of bias assessment of the included studies. Note: Red color: high risk; Yellow color: unclear risk; Green color: low risk.

All 32 studies mentioned the use of random allocation methods, with 18 studies using random number tables, 13 studies using simple randomization, and one study using sequential allocation. Two studies employed double-blinding, and two studies employed single-blinding, while the remaining 28 studies did not mention the use of blinding. The outcome data of all included studies were relatively complete, with no bias due to incomplete outcome reporting, and the reported methods and results were consistent. Other sources of bias were unclear. The risk of bias assessment results for the included studies are illustrated in Figure 2.

### 3.4 Efficacy for outcome measures

Heterogeneity analysis using the “mtc.anohe” function showed that heterogeneity did not significantly affect the results overall. The consistency hypothesis using the node-splitting method showed that all *p*-values were ≥0.05, indicating no significant inconsistency. Convergence of the iterations was quantified by calculating the PSRF, and a PSRF value of 1.00 indicated satisfactory convergence. In conclusion, conducting network meta-analysis for these outcome measures was reasonable.

#### 3.4.1 Cardiac index

Regarding the meta-analysis of CI level, a total of 17 studies with 1,414 patients were included. Four different interventions were involved, with levosimendan having two different doses of 0.1 µg/kg/min and 0.2 µg/kg/min. Three studies only used a placebo intervention, 14 studies used dobutamine intervention with no specific dose, four studies used levosimendan intervention at a dose of 0.1 µg/kg/min, and 13 studies used levosimendan intervention at a dose of 0.2 µg/kg/min (Supplementary Figure SI-1 in Supplementary Material S1).

In comparison to the placebo group, dobutamine (MD [95% CrI] = 0.014 [−0.43, 0.47]) did not show a significant advantage in improving CI. However, levosimendan, at 0.1 µg/kg/min (MD [95% CrI] = 0.41 [-0.43, 1.4]) and 0.2 µg/kg/min (MD [95% CrI] = 0.54 [0.12, 0.99]), significantly improved the CI levels of patients. (Supplementary Figure SI-3.1 in Supplementary Material S1). Additionally, it was observed that levosimendan at 0.1 µg/kg/min was superior to 0.2 µg/kg/min (MD [95% CrI] = 0.34 [−0.06, 0.72) (Supplementary Table SI-3.2 in Supplementary Material S1). The probability ranking based on SUCRA values showed a consistent trend with the forest plot and league table: dobutamine (17.61%), levosimendan at 0.1 µg/kg/min (98.57%), levosimendan at 0.2 µg/kg/min (67.80%), and placebo (16.02%). According to the SUCRA values, levosimendan 0.1 µg/kg/min was superior to levosimendan 0.2 µg/kg/min, with a significant difference. Therefore, levosimendan at 0.1 µg/kg/min may be preferred in clinical practice. The reason for this difference may be related to the small number of included studies and the limited evidence base.

#### 3.4.2 Lactic acid

Twenty-one studies reported Lac levels, including 1,605 patients. There were six interventions, including three different doses of levosimendan: 0.075 µg/kg/min, 0.1 µg/kg/min, and 0.2 µg/kg/min. Three studies used placebo as the intervention, 16 studies used dobutamine with no specific dose, two studies used milrinone with no specific dose, two studies used levosimendan at a dose of 0.075 µg/kg/min, five studies used levosimendan at a dose of 0.1 µg/kg/min, and 14 studies used levosimendan at a dose of 0.2 µg/kg/min (Supplementary Figure SII-1 in Supplementary Material S1).

Compared to the placebo group, dobutamine (MD [95% CrI] = 1.3 [0.94, 1.6]) significantly increased Lac levels. Additionally, milrinone (MD [95% CrI] = 0.43 [−0.84, 1.7]) showed a trend of increasing Lac levels, but this trend was not statistically significant due to the wide width of the 95% CrI, which included the null value. Levosimendan, at both 0.075 µg/kg/min (MD [95% CrI] = 0.033 [−0.75, 0.82]) and 0.2 µg/kg/min (MD [95% CrI] = −0.014 [−0.26, 0.23]), did not show a significant advantage in improving Lac. However, levosimendan at 0.1 µg/kg/min (MD [95% CrI] = −0.47 [−0.90, −0.043]) significantly reduced Lac levels in patients (Supplementary Figure SII-3.1 in Supplementary Material S1). In addition, according to the league table, levosimendan at 0.1 µg/kg/min showed a significant superiority over dobutamine (MD [95% CrI] = 1.72 [1.4, 2.04]) (Supplementary Table SII-3.2 in Supplementary Material S1). The probability rankings based on SUCRA values reflected a consistent trend with the forest plot and league table: dobutamine (97.98%), milrinone (65.04%), levosimendan at 0.075 µg/kg/min (44.92%), levosimendan at 0.1 µg/kg/min (4.34%), levosimendan at 0.2 µg/kg/min (42.73%), and placebo (45.00%).

#### 3.4.3 28-day mortality

Twenty-two studies reported 28-day mortality rates, including 2,130 patients. There are 6 intervention measures involved, among which levosimendan has three different doses: 0.075 µg/kg/min, 0.1 µg/kg/min, and 0.2 µg/kg/min. Five studies used a placebo as the intervention, fifteen studies used dobutamine, two studies used milrinone, two studies used levosimendan at a dose of 0.075 µg/kg/min, six studies used levosimendan at a dose of 0.1 µg/kg/min, and fourteen studies used levosimendan at a dose of 0.2 µg/kg/min (Supplementary Figure SIII-1 in Supplementary Material S1).

Compared to the placebo group, dobutamine (RR [95% CrI] = 1.3 [1.0, 1.7]) was associated with an increased 28-day mortality rate, while milrinone (RR [95% CrI] = 1.5 [0.65, 3.6]) showed a trend towards increasing the mortality, although the wide 95% CrI included the null value, making this trend non-significant. Levosimendan, at 0.1 µg/kg/min (RR [95% CrI] = 0.99 [0.73, 1.3]) and 0.2 µg/kg/min (RR [95% CrI] = 1.0 [0.88, 1.2]), did not show significant advantages in improving mortality rates. Levosimendan at 0.075 µg/kg/min (RR [95% CrI] = 0.72 [0.30, 1.6]) showed a trend towards reducing the 28-day mortality rate. However, this trend was not statistically significant due to the wide 95% CrI, which included the null value (Supplementary Figure SIII-3.1 in Supplementary Material S1). Additionally, the league table showed that levosimendan at 0.075 µg/kg/min was superior to milrinone (RR [95% CrI] = 2.07 [0.77, 6.18)] (Supplementary Table SIII-3.2 in Supplementary Material S1). The SUCRA rankings align with the trends observed in the forest plot and league table: dobutamine (15.43%), milrinone (19.31%), levosimendan at a dose of 0.075 µg/kg/min (84.05%), levosimendan at a dose of 0.1 µg/kg/min (63.69%), levosimendan at a dose of 0.2 µg/kg/min (53.94%), and placebo (63.57%). Based on the SUCRA values, there is a substantial difference between levosimendan at 0.075 µg/kg/min and dobutamine, as well as milrinone. Therefore, the clinical use of levosimendan at 0.075 µg/kg/min may lead to greater benefits for patients. However, the observed differences did not reach statistical significance as levosimendan at 0.075 µg/kg/min was only reported in two studies, and there was heterogeneity within these studies.

## 4 Discussion

The results of this network meta-analysis showed that levosimendan significantly improved CI levels and Lac levels in patients with sepsis. All interventions in this study failed to reduce 28-day mortality. Levosimendan 0.1 µg/kg/min may be the optimal dose to improve CI levels and Lac levels in patients with sepsis.

Dobutamine, milrinone, and levosimendan all have positive effects on heart muscle contraction, but they work through different mechanisms. Dobutamine selectively activates the heart β_1_ receptor, increasing the concentration of calcium ions inside the heart muscle cells, thereby enhancing myocardial contractility and significantly reducing the levels of plasma cTnI and heart-type fatty acid binding protein (H-FABP) (Stratton et al., 2017). Milrinone can improve cardiac function (Wang et al., 2021; Zhuang et al., 2023) by inhibiting the intracellular nucleotide phosphodiesterase pathway, increasing the concentration of intracellular cyclic adenosine monophosphate (cAMP) (Dünser et al., 2009), enhancing the release of intracellular calcium, relaxing vascular smooth muscle (Werner et al., 1995; Zhao et al., 2021) and reducing the expression of inflammatory factors (Kern et al., 2001). However, both dobutamine and milrinone have limitations as they increase myocardial oxygen consumption, posing a risk of adverse events (Schmittinger et al., 2012; Chang et al., 2020), and are thus not recommended for long-term clinical use (Fang et al., 2021). Levosimendan, on the other hand, improves myocardial contractility by stabilizing the “cTnC-Ca^2+^” complex without increasing intracellular calcium concentration and cell oxygen consumption (Ponikowski et al., 2016). Levosimendan also directly activates eNOS on endothelial cells, leading to increased production of nitric oxide (NO) (Pataricza et al., 2003), as well as indirectly activate K_ATP_ channels and Ca^2+^and voltage-sensitive K^+^ channels on vascular smooth muscle cells through modulation of certain signaling molecules (Grossini et al., 2009). This leads to the dilation of coronary and systemic blood vessels, thus improving myocardial ischemia (Pollesello et al., 2016). Additionally, levosimendan has the ability to suppress inflammatory reactions and myocardial apoptosis, as well as to combat oxidative damage. It reduces the release of oxidative markers (such as TBARS), increases plasma GSH levels (Grossini et al., 2020), and enhances cell autophagy (Schellekens et al., 2015), which plays a role in early-stage cell repair (Schauer et al., 2021) and long-term heart protection (Paraskevaidis et al., 2005). Furthermore, it has advantages in improving cellular metabolism in septic patients and provides certain protective effects on lung, kidney (Lannemyr et al., 2018), and diaphragm function (Doorduin et al., 2012).

We found that levosimendan significantly increased CI in patients with sepsis, and different doses also affected the extent of the increase in CI. Currently, the optimal dose of levosimendan is still controversial. Our results suggest that levosimendan 0.1 µg/kg/min may benefit patients more. Levosimendan has a short half-life of only 1–1.5 h, with a plasma protein binding rate of 95%, and is metabolized by the liver and intestine into OR-1855 and OR-1896. OR-1855 is inactive, while OR-1896 has a structure and function similar to levosimendan, with a half-life of nearly 77 h (Banfor et al., 2008). The typical loading dose for levosimendan is 6–24 μg/kg, with a maintenance dose starting at 0.1 μg/kg/min, which can be adjusted based on the situation within the range of 0.05–0.2 μg/kg/min (Leppikangas et al., 2011). To avoid sudden drop in blood pressure, worsening of coronary ischemia, and arrhythmias caused by the loading dose, it may be advisable to avoid using the loading dose or to use other positive inotropic and vasopressor drugs concurrently (Mehta et al., 2017; Herpain et al., 2019). Hence, we believe that recommending a dosage of 0.1 μg/kg/min for levosimendan is reasonable. The improvement in CI by levosimendan is mainly mediated through its calcium sensitization effect while selectively inhibiting phosphodiesterase III (PDE III) isoform and activating potassium channels. During septic shock, there is a significant downregulation of L-type calcium channels in myocardial cell membranes, leading to decreased sensitivity of myofilaments to calcium ions (Lew et al., 1996; Tavernier et al., 2001; Hobai et al., 2015). On the one hand, levosimendan and its metabolite OR-1896 directly bind to troponin C in cardiomyocytes, improving the stability of myocardial fibrin spatial configuration and calcium myofilament reactivity, thus increasing myocardial contractility (Sorsa et al., 2004; Zhang et al., 2015). Levosimendan, on the other hand, selectively inhibits PDEⅢ isoform activity, increases cAMP levels, promotes voltage-gated calcium channel phosphorylation, and allows calcium ions to enter the cell, showing additional positive inotropic effects (Szilágyi et al., 2004). Finally, levosimendan can also increase the outward potassium (K^+^) current by activating the adenosine triphosphate-dependent K^+^ channels, leading to membrane hyperpolarization. This causes the closure of voltage-dependent calcium (Ca^2+^) channels on the cellular membrane, effectively inhibiting the influx of Ca^2+^. Additionally, it activates the sodium-calcium exchange channels, promoting the efflux of Ca^2+^ and consequently reducing the intracellular Ca^2+^ concentration (Yokoshiki et al., 1997). At the same time, it opens the potassium channel of vascular smooth muscle, thus dilating blood vessels, reducing cardiac preload, increasing myocardial contractility and coronary blood supply, and improving heart pump function (Zangrillo et al., 2015). While levosimendan increases the CI, it does not increase myocardial oxygen consumption (Zhang et al., 2015). This may be attributed to its positive inotropic effect, which is not dependent on membrane depolarization-induced intracellular calcium influx, thereby avoiding intracellular calcium overload (Kaheinen et al., 2004). Maack et al. (Maack et al., 2019) proposed that the positive inotropic effect of levosimendan was a result of the synergistic action of PDEⅢ inhibition and calcium sensitization, with the latter being more important. This also explains why levosimendan, when exerting its positive inotropic effect through PDEⅢ inhibition, does not lead to intracellular calcium overload. However, there is currently limited research to demonstrate whether different dosing regimens of levosimendan have varying effects on CI levels and blood lactate levels. Therefore, multicenter, prospective RCTs are required to determine the optimal dosing regimen, efficacy, and safety of levosimendan in sepsis.

This study demonstrated that levosimendan could reduce Lac levels in patients with sepsis by improving microcirculation. The mechanisms of the improved microcirculation may be mainly related to vasodilation, anti-inflammatory, and anti-oxidative effects (Parissis et al., 2004; Wang et al., 2015; Hajjej et al., 2017; Heringlake et al., 2021). Levosimendan induces vasodilation by promoting the opening of ATP-sensitive potassium channels (K^+^_ATP_ channels) in vascular smooth muscle cells, thereby dilating afferent arterioles in the kidney, increasing renal blood flow, and improving lactate clearance in septic patients with liver and kidney dysfunction (Heringlake et al., 2021; Vincent and Bakker, 2021). Some studies have found that levosimendan can downregulate NF-κB-dependent transcription, inhibit inducible nitric oxide (NO) synthase promoter activity, and reduce NO expression (Sareila et al., 2008), thereby reducing the levels of inflammatory factors and oxidative products such as brain natriuretic peptide (BNP), interleukin-6 (IL-6), tumor necrosis factor-alpha (TNF-α), and C-reactive protein (CRP) in patients with sepsis (Farmakis et al., 2016). This suggests that levosimendan exerts anti-inflammatory and antioxidant effects (Parissis et al., 2004; Wang et al., 2015; Hajjej et al., 2017). These findings indirectly demonstrate that levosimendan can improve tissue microcirculatory ischemia and hypoxia, alleviate mitochondrial dysfunction, reduce lactate levels, improve organ microcirculatory perfusion (Morelli et al., 2006), and further validate the effectiveness of levosimendan in improving hemodynamics in septic patients (Memiş et al., 2012).

This study found that, like other drugs analyzed, levosimendan did not reduce patient mortality, and there is currently no consensus on this phenomenon. A meta-analysis conducted by Zangrillo et al. (2015) on the effect of levosimendan on mortality in septic shock patients showed a reduction in mortality. However, studies by Bhattacharjee et al. (2017) and Chang et al. (2018) both indicated that levosimendan did not reduce mortality in sepsis and septic shock patients. In this study, none of the interventions, including levosimendan, were able to reduce the 28-day mortality rate, and the use of dopamine and norepinephrine increased the risk of 28-day mortality. The inability of levosimendan to reduce mortality rates may be related to various complex factors influencing mortality. Although we found that levosimendan can increase cardiac index and reduce blood lactate levels, these benefits did not translate into clinical endpoints. Some studies suggest that levosimendan can increase heart rate, thereby increasing the possibility of tachyarrhythmias, which is unfavorable for septic patients (Barraud et al., 2007). The main reasons for levosimendan-induced tachycardia are twofold. On one hand, it is due to the vasodilatory effects of levosimendan. On the other hand, Gordon et al. found that patients on levosimendan had a higher requirement for norepinephrine, which may contribute to tachycardia (Barraud et al., 2007; Torraco et al., 2014). Therefore, the use of rate-control medications in sepsis patients with co-existing tachycardia may be beneficial (Fang and Dong, 2014). Although guidelines suggest the use of dobutamine in sepsis patients with inadequate perfusion, there is a lack of definitive evidence supporting this suggestion (Sato and Nasu, 2015). As a result, it remains unclear whether positive inotropes offer any additional benefits to sepsis compared to norepinephrine.

The inability of levosimendan to reduce mortality rates may be attributable to its adverse reactions. Common adverse effects of levosimendan include hypotension, arrhythmias, and decreased red blood cell volume (Wang and Wang, 2019). Levosimendan increases positive inotropy by increasing calcium sensitivity without increasing myocardial oxygen consumption, differing in pharmacological mechanism from catecholamine-based positive inotropic drugs that act through beta-adrenergic receptors. Therefore, theoretically, the incidence of arrhythmias with levosimendan in clinical use should be relatively reduced compared to other positive inotropic drugs. However, in practical application, levosimendan’s adverse reactions related to arrhythmias do not show a significant decrease (Maack et al., 2019). Basic research suggested that levosimendan induced atrial fibrillation by shortening action potential duration and refractoriness, leading to atrial tissue activation and enhanced electrical circuit activity (Frommeyer et al., 2017). Follath et al. (2002) indicated that the decrease in red blood cell volume was attributable to the vasodilatory effect of levosimendan, leading to blood dilution. Sepsis patients often have unstable hemodynamics and are at risk of hypotension; the vasodilatory effect of levosimendan further increases this risk. Therefore, levosimendan is recommended for patients who have undergone adequate fluid resuscitation and have achieved the target mean arterial pressure (Society of Critical Care Medicine and Chinese Medical Association, 2015).

This study has several limitations. Firstly, although we have included all relevant studies to date, the sample size is still relatively small. Apart from the studies by Gordon et al. (2016) and Janssens (2017), which had over 500 cases, all other studies included fewer than 150 cases. Secondly, this study treated severe sepsis and septic shock patients as a whole, which may lead to heterogeneity. Owing to the limited number of studies available, we were unable to conduct subgroup analysis on different patient groups. Thirdly, some studies reported varying loading doses of levosimendan, and the duration of drug use varied among studies. However, due to the limited number of studies, subgroup analyses could not be carried out. Lastly, none of the included studies provided information stratified by sex; therefore, we did not explore the influence of sex-related factors. Future research should explore the impact of factors such as sex and age on the efficacy of levosimendan in clinical use.

## 5 Conclusion

Based on existing evidence, it can be concluded that levosimendan does not reduce mortality but can improve the CI and reduce blood Lac levels in sepsis patients. Levosimendan at 0.1 µg/kg/min is recommended. Hence, levosimendan has certain clinical value in the management of sepsis. Nevertheless, given the overall low quality and insufficient sample size of the included studies, these findings are insufficient to guide clinical practice, and physicians should exercise caution when interpreting these findings. Furthermore, more high-quality research is needed to further explore the efficacy of levosimendan in the treatment of sepsis and related conditions.

## Data Availability

The original contributions presented in the study are included in the article/Supplementary Material, further inquiries can be directed to the corresponding author.
