# Supplementary material for: Efficacy and safety of levosimendan in patients with sepsis: a systematic review and network meta-analysis
Source: Front Pharmacol. 2024 Mar 8;15:1358735. doi: 10.3389/fphar.2024.1358735 (PMC10957638; doi:10.3389/fphar.2024.1358735)
Supplement: Supplementary file 1 [file DataSheet2.docx]

**Efficacy and safety of levosimendan in patients with sepsis: a systematic review and network meta-analysis**

**Ruimin Tan, He Guo, Zinan Yang, HuiHui Yang, Qinghao Li, Qiong Zhu, Quansheng Du^*^**

***** **Correspondence:** Quansheng Du

Address: No. 348 Heping West Road,Xinhua District, Shijiazhuang City, Hebei Province, the People's Republic of China.

E-mail: dqs888@126.com

Phone number: +86 13230163769

**Supplementary Material 2** Search strategy and result

| PubMed | | | | |
| --- | --- | --- | --- | --- |
| No. | Query | | | Hits |
| #5 | (#1 OR #2) AND (#3 OR #4) | | | 142 |
| #4 | Simendan | | | 1318 |
| #3 | "daxim" OR "Dextrosimendan" OR "levosimedan" OR "Levosimendan" OR "odm 109" OR "OR 1259" OR "OR 1855" OR "Simadax" OR "simdax" OR "Simendan" | | | 1704 |
| #2 | Sepsis | | | 142904 |
| #1 | "abdominal sepsis" OR "Blood Poisoning*" OR "Bloodstream Infection*" OR "focal sepsis" OR "intraabdominal sepsis" OR "Pyaemia*" OR "Pyemia*" OR "Pyohemia*" OR "Sepsis" OR "sepsis syndrome" OR "septic disease" OR "Septicemia*" OR "Severe Sepsis" | | | 150452 |
| Web of Science | | | | |
| #1 | (TI=(("abdominal sepsis") OR ("Blood Poisoning") OR ("Bloodstream Infection") OR ("focal sepsis") OR ("intraabdominal sepsis") OR (Pyaemia*) OR (Pyemia*) OR (Pyohemia*) OR ("Sepsis") OR ("sepsis syndrome") OR ("septic disease") OR (Septicemia*) OR ("Severe Sepsis")) OR AB=(("abdominal sepsis") OR ("Blood Poisoning") OR ("Bloodstream Infection") OR ("focal sepsis") OR ("intraabdominal sepsis") OR (Pyaemia*) OR (Pyemia*) OR (Pyohemia*) OR ("Sepsis") OR ("sepsis syndrome") OR ("septic disease") OR (Septicemia*) OR ("Severe Sepsis")) OR AK=(("abdominal sepsis") OR ("Blood Poisoning") OR ("Bloodstream Infection") OR ("focal sepsis") OR ("intraabdominal sepsis") OR (Pyaemia*) OR (Pyemia*) OR (Pyohemia*) OR ("Sepsis") OR ("sepsis syndrome") OR ("septic disease") OR (Septicemia*) OR ("Severe Sepsis"))) AND (TI=(("daxim") OR ("Dextrosimendan") OR ("levosimedan") OR ("Levosimendan") OR ("odm 109") OR ("OR 1259") OR ("OR 1855") OR ("Simadax") OR ("simdax") OR ("Simendan")) OR AB=(("daxim") OR ("Dextrosimendan") OR ("levosimedan") OR ("Levosimendan") OR ("odm 109") OR ("OR 1259") OR ("OR 1855") OR ("Simadax") OR ("simdax") OR ("Simendan")) OR AK=(("daxim") OR ("Dextrosimendan") OR ("levosimedan") OR ("Levosimendan") OR ("odm 109") OR ("OR 1259") OR ("OR 1855") OR ("Simadax") OR ("simdax") OR ("Simendan"))) | | | 112 |
| Embase | | | | |
| #5 | (#1 OR #2) AND (#3 OR #4) | | | 542 |
| #4 | 'levosimendan'/exp | | | 4563 |
| #3 | ('daxim' OR 'Dextrosimendan' OR 'levosimedan' OR 'Levosimendan' OR 'odm 109' OR 'OR 1259' OR 'OR 1855' OR 'Simadax' OR 'simdax' OR 'Simendan'):ti,ab,kw | | | 2948 |
| #2 | 'sepsis'/exp | | | 353965 |
| #1 | ('abdominal sepsis' OR 'Blood Poisoning*' OR 'Bloodstream Infection*' OR 'focal sepsis' OR 'intraabdominal sepsis' OR 'Pyaemia*' OR 'Pyemia*' OR 'Pyohemia*' OR 'Sepsis' OR 'sepsis syndrome' OR 'septic disease' OR 'Septicemia*' OR 'Severe Sepsis'):ti,ab,kw | | | 233900 |
| Cochrane | | | | |
| #5 | (#1 OR #2) AND (#3 OR #4) | | | 65 |
| #4 | Simendan | | | 320 |
| #3 | ('daxim' OR 'Dextrosimendan' OR 'levosimedan' OR 'Levosimendan' OR 'odm 109' OR 'OR 1259' OR 'OR 1855' OR 'Simadax' OR 'simdax' OR 'Simendan'):ti,ab,kw | | | 2254 |
| #2 | Sepsis | | | 6837 |
| #1 | ('abdominal sepsis' OR 'Blood Poisoning*' OR 'Bloodstream Infection*' OR 'focal sepsis' OR 'intraabdominal sepsis' OR 'Pyaemia*' OR 'Pyemia*' OR 'Pyohemia*' OR 'Sepsis' OR 'sepsis syndrome' OR 'septic disease' OR 'Septicemia*' OR 'Severe Sepsis'):ti,ab,kw | | | 16692 |
| CNKI | | | | |
| #1 | | (篇关摘%脓毒 + 脓毒症 + 脓毒性休克 + 感染性休克) AND (篇关摘%左西孟旦) 资源范围:总库；中英文扩展；更新时间:不限 | 89 | |
| Wanfang data | | | | |
| #1 | | (题名或关键词%脓毒 or 脓毒症 or 脓毒性休克 or 感染性休克) AND (题名或关键词%左西孟旦) | 101 | |
| VIP | | | | |
| #1 | | (题名或关键词%脓毒 + 脓毒症 + 脓毒性休克 + 感染性休克) AND (题名或关键词%左西孟旦) | 82 | |
| CBM | | | | |
| #1 | | (全部字段%脓毒 OR 脓毒症 OR 脓毒性休克 OR 感染性休克) AND (全部字段%左西孟旦) | 83 | |
