# Supplementary material for: Efficacy and safety of levosimendan in patients with sepsis: a systematic review and network meta-analysis
Source: Front Pharmacol. 2024 Mar 8;15:1358735. doi: 10.3389/fphar.2024.1358735 (PMC10957638; doi:10.3389/fphar.2024.1358735)
Supplement: Supplementary file 2 [file DataSheet1.docx]

**Efficacy and safety of levosimendan in patients with sepsis: a systematic review and network meta-analysis**

**Ruimin Tan, He Guo, Zinan Yang, HuiHui Yang, Qinghao Li, Qiong Zhu, Quansheng Du^*^**

***** **Correspondence:** Quansheng Du

Address: No. 348 Heping West Road,Xinhua District, Shijiazhuang City, Hebei Province, the People's Republic of China.

E-mail: dqs888@126.com

Phone number: +86 13230163769

Supplementary Material 1 Statistical Analysis Protocol and Results

**I Cardiac index**

In this part, we used "gemtc" package(version 1.0-1) in R(version 4.2.3) to conduct the Bayesian Meta analysis.

1. **Building Network**
   1. **Function used:** *mtc.network*
   2. **Results:**


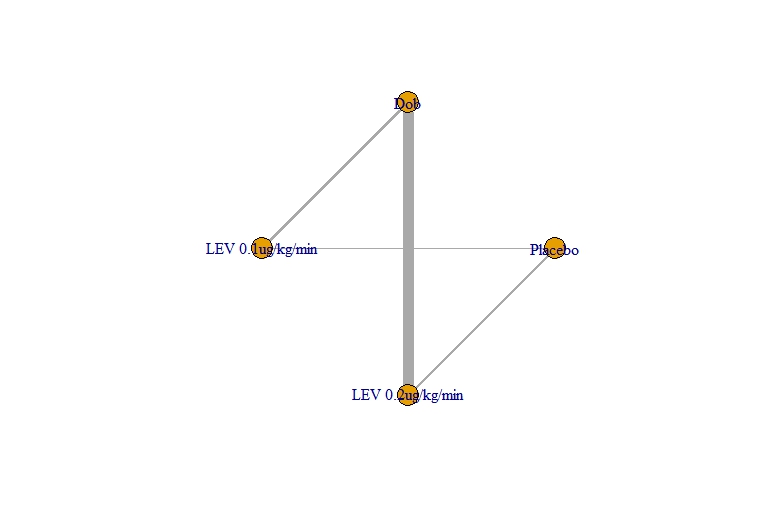


**Figure SI-1 Network of Cardiac index**

1. **Modelling and Running**
   1. **Function used:** *mtc.model, mtc.run*
2. **Results Presentation:**
   1. **Forest plot (Placebo was set as the baseline to calculate the relative effect among arms)**


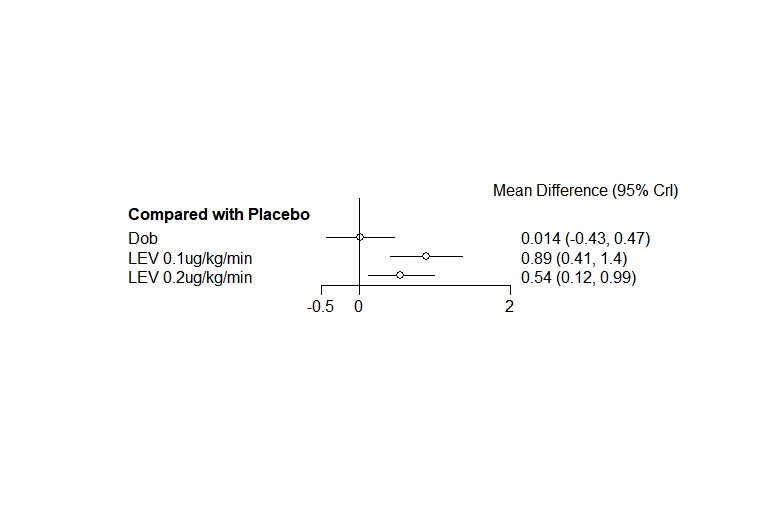


**Figure SI-3.1 Relative Effect Forest Plot of Cardiac index (baseline: placebo)**

**Abbreviation:** CrI, credible interval.

- 1. **League table**

**Table SI-3.2 League Table of Cardiac index**

| Placebo |  |  |  |
| --- | --- | --- | --- |
| -0.01 (-0.47, 0.43) | Dob |  |  |
| **-0.89 (-1.36, -0.41)** | **-0.87 (-1.22, -0.52)** | LEV 0.1ug/kg/min |  |
| **-0.54 (-0.99, -0.12)** | **-0.53 (-0.74, -0.33)** | 0.34 (-0.06, 0.72) | LEV 0.2ug/kg/min |

- 1. **Rank of probability**


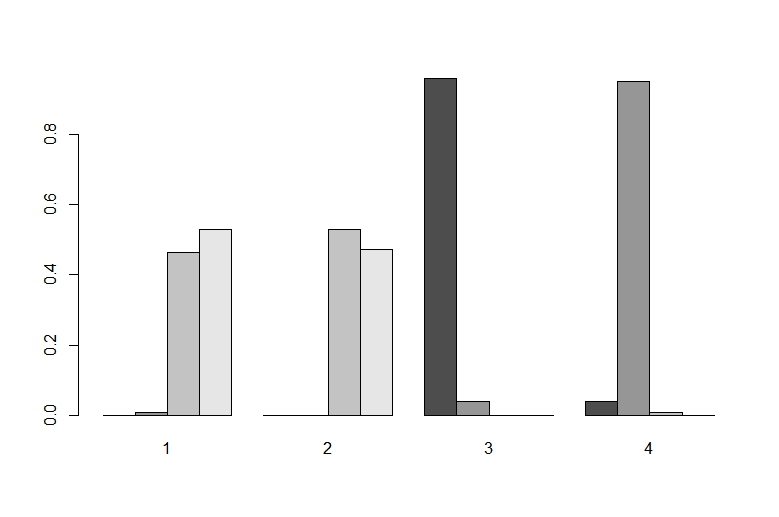


**Figure SI-3.3 Bar chart of rank of probability of cardiac index.**

**Notes:** 1, Placebo; 2, Dob; 3, LEV 0.1ug/kg/min;4, LEV 0.2ug/kg/min.

- 1. **SUCRA rank of probability**

**Table SI-3.4 SUCRA rank of probability of cardiac index.**

| Intervention | SUCRA |
| --- | --- |
| Placebo | 16.02% |
| Dob | 17.61% |
| LEV 0.1ug/kg/min | 98.57% |
| LEV 0.2ug/kg/min | 67.80% |

**Abbreviation:** SUCRA, surface area under the cumulative ranking curve.

1. **Quality Control**
   1. **Convergence test: PSRF value**


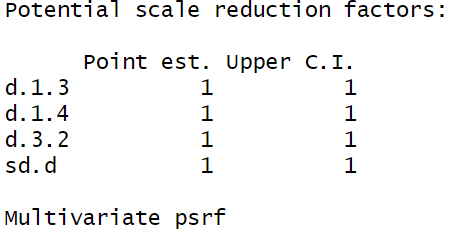


**Figure SI-4.1 PSRF of the Bayesian analysis of cardiac index**

**Abbreviation:** PSRF, Potential scale reduction factors.

**Note:** The closer the PSRF value is to 1, the better. >1.05 indicates unsatisfactory convergence.

- 1. **Consistency hypothesis: inconsistency test.**
     1. **Function used:** *mtc.nodesplit*
     2. **Results:**

**
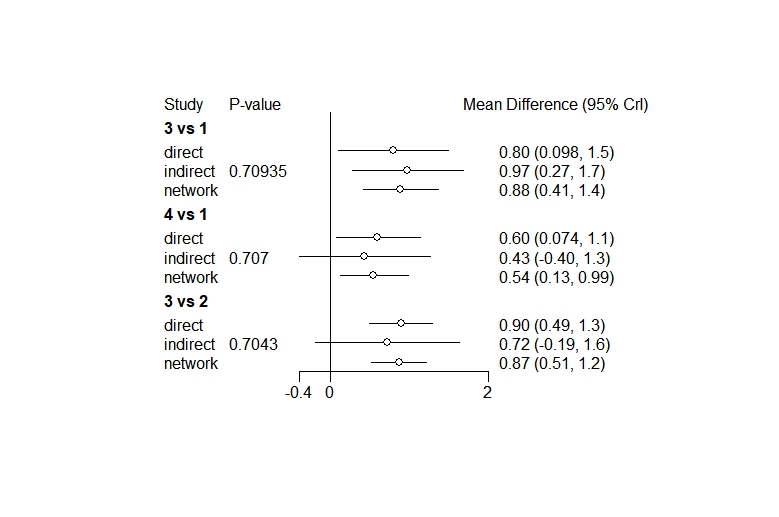

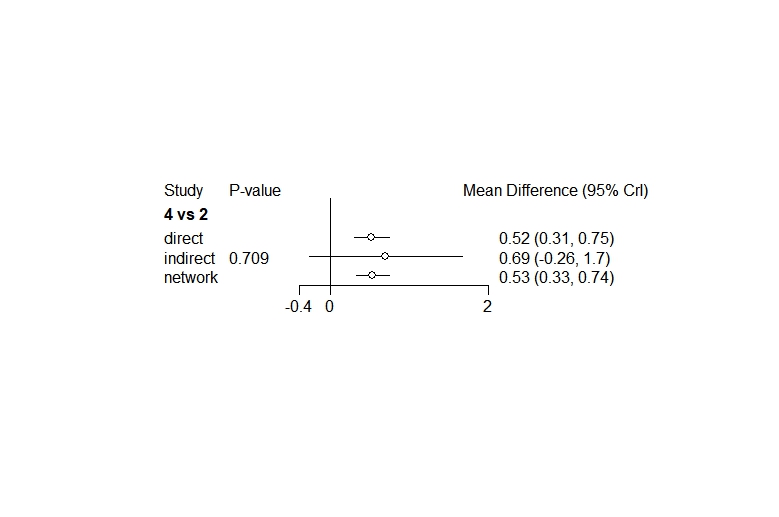
**

**Figure SI-4.2.2 Inconsistency test of the Bayesian analysis of cardiac index**

**Note:** 1, Placebo; 2, Dob; 3, LEV 0.1ug/kg/min;4, LEV 0.2ug/kg/min. *P* value > 0.05 indicates that there is no significant inconsistency which means the direct, indirect and network comparison is consistent.

- 1. **Homogeneity hypothesis: Heterogeneity analysis**
     1. **Function used:** *mtc.anohe*
     2. **Results**


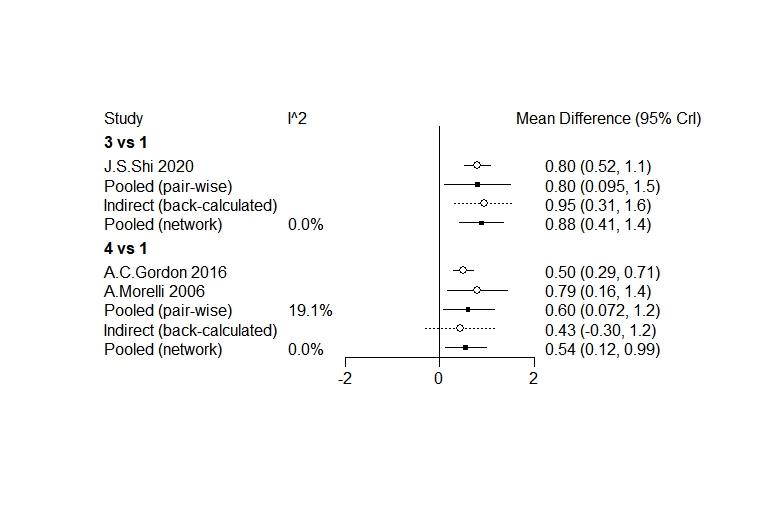

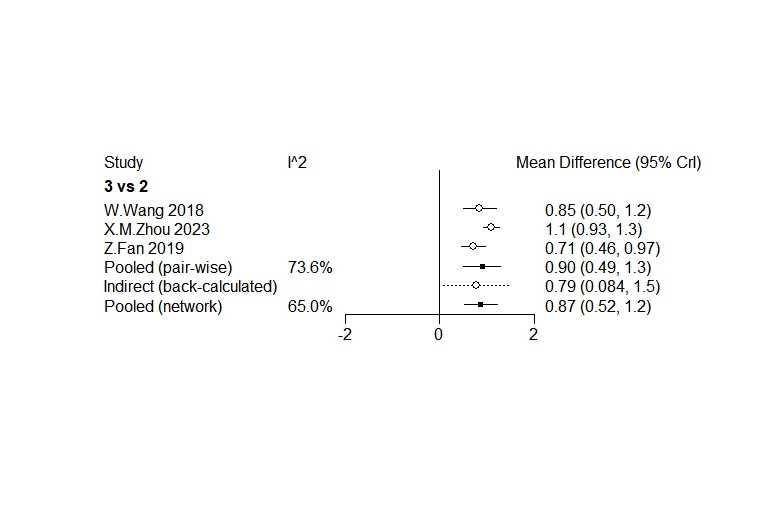

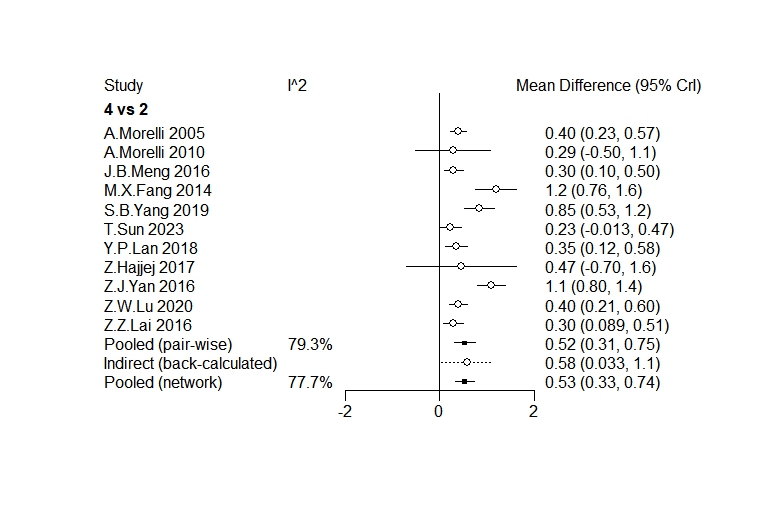


**Figure SI-4.3.2 Heterogeneity analysis of the Bayesian analysis of cardiac index**

**Note:** From the figure we could see, there exists no significant heterogeneity between any two arms, which allows the use of fixed effect model to estimate the pooling results.

**II Lactic acid**

In this part, we used "gemtc" package(version 1.0-1) in R(version 4.2.3) to conduct the Bayesian Meta analysis.

1. **Building Network**
   1. **Function used:** *mtc.network*
   2. **Results:**


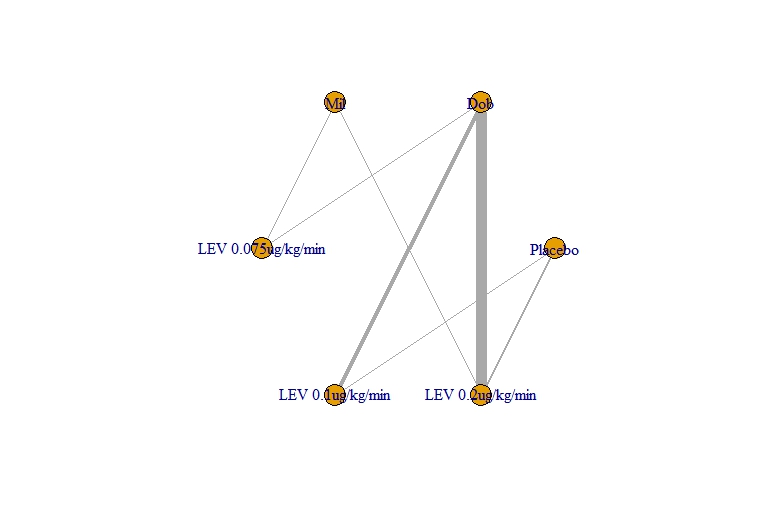


**Figure SII-1 Network of Lactic acid**

1. **Modelling and Running**
   1. **Function used:** *mtc.model, mtc.run*
2. **Results Presentation:**
   1. **Forest plot (Placebo was set as the baseline to calculate the relative effect among arms)**


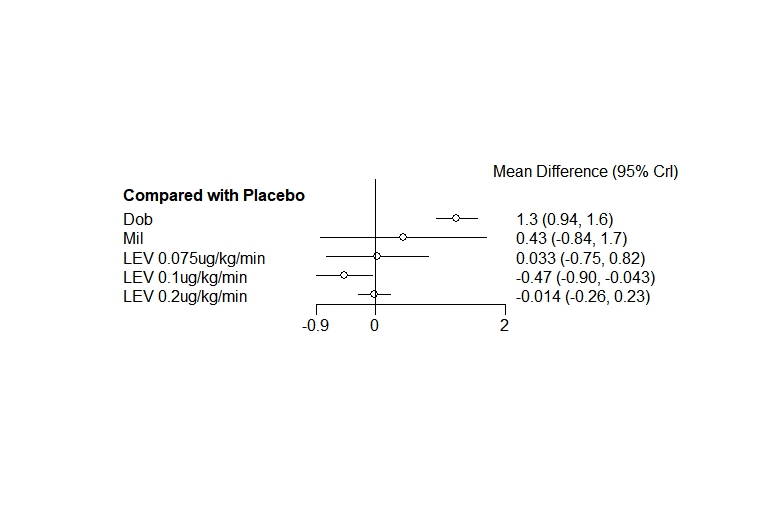


**Figure SII-3.1 Relative Effect Forest Plot of Lactic acid**

**Abbreviation:** CrI, credible interval.

- 1. **League table**

**Table SII-3.2 League Table of Lactic acid**

| Placebo |  |  |  |  |  |
| --- | --- | --- | --- | --- | --- |
| **-1.25 (-1.57, -0.94)** | Dob |  |  |  |  |
| -0.43 (-1.71, 0.84) | 0.82 (-0.44, 2.08) | Mil |  |  |  |
| -0.03 (-0.82, 0.75) | **1.22 (0.5, 1.94)** | 0.4 (-0.88, 1.68) | LEV 0.075ug/kg/min |  |  |
| **0.47 (0.04, 0.9)** | **1.72 (1.4, 2.04)** | 0.9 (-0.39, 2.2) | 0.5 (-0.29, 1.29) | LEV 0.1ug/kg/min |  |
| 0.01 (-0.23, 0.26) | **1.27 (1.05, 1.49)** | 0.44 (-0.8, 1.7) | 0.05 (-0.7, 0.8) | -0.45 (-0.83, -0.08) | LEV 0.2ug/kg/min |

- 1. **Rank of probability**


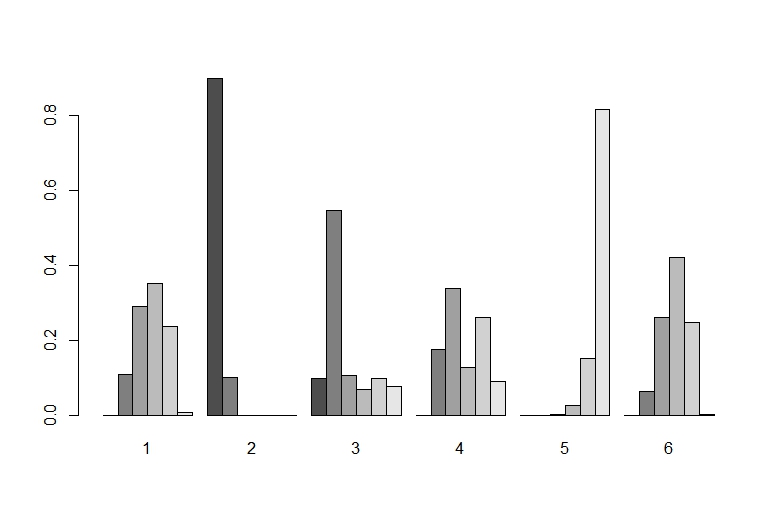


**Figure SII-3.3 Bar chart of rank of probability of lactic acid.**

**Notes:** 1, Placebo; 2, Dob; 3, Mil; 4, LEV 0.075ug/kg/min;5, LEV 0.1ug/kg/min;6, LEV 0.2ug/kg/min.

- 1. **SUCRA rank of probability**

**Table SII-3.4 SUCRA rank of probability of lactic acid.**

| Intervention | SUCRA |
| --- | --- |
| Placebo | 45.00% |
| Dob | 97.98% |
| Mil | 65.04% |
| LEV 0.075ug/kg/min | 44.92% |
| LEV 0.1ug/kg/min | 4.34% |
| LEV 0.2ug/kg/min | 42.73% |

**Abbreviation:** SUCRA, surface area under the cumulative ranking curve.

1. **Quality Control**
   1. **Convergence test: PSRF value**


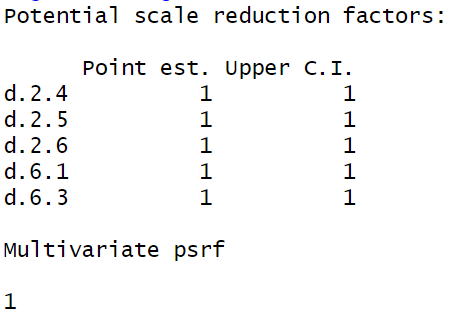


**Figure SII-4.1 PSRF of the Bayesian analysis of lactic acid**

**Abbreviation:** PSRF, Potential scale reduction factors.

**Note:** The closer the PSRF value is to 1, the better. >1.05 indicates unsatisfactory convergence.

- 1. **Consistency hypothesis: inconsistency test.**
     1. **Function used:** *mtc.nodesplit*
     2. **Results:**

**
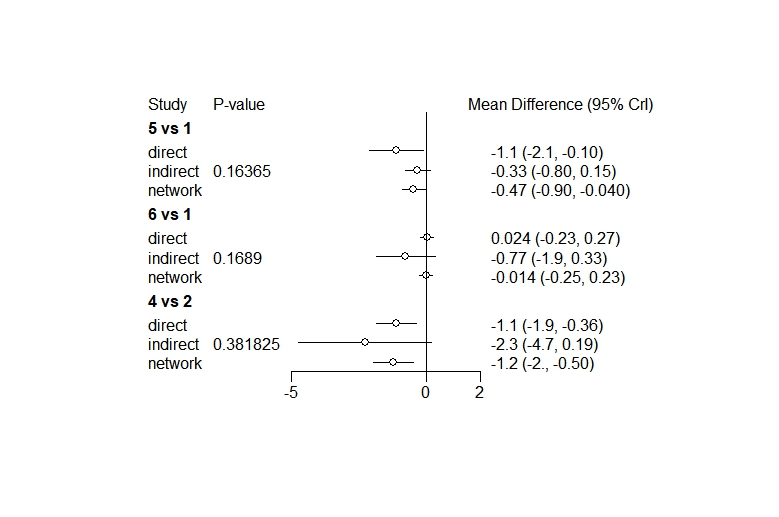
**

**
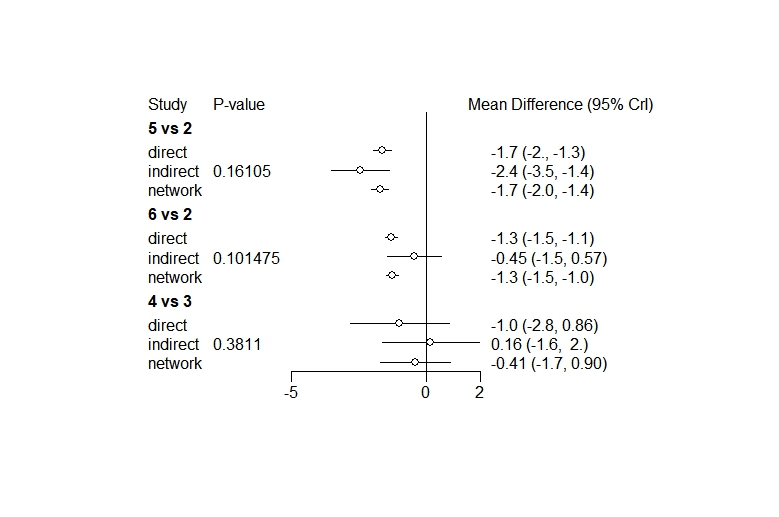
**

**
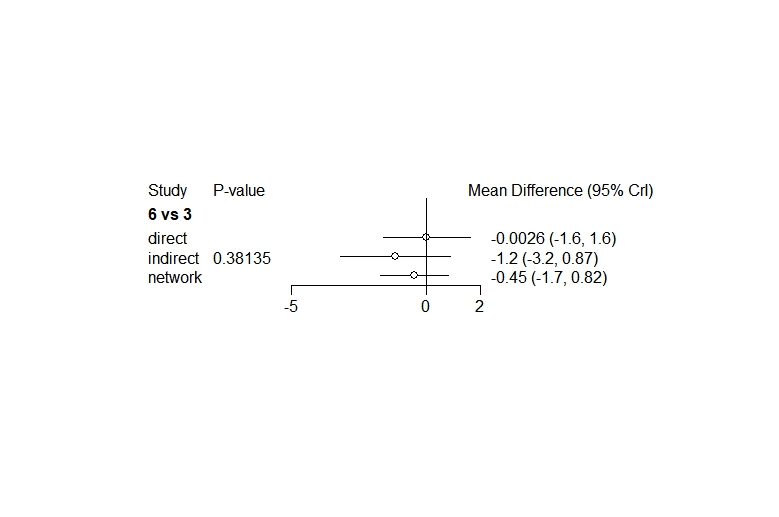
**

**Figure SII-4.2.2 Inconsistency test of the Bayesian analysis of lactic acid**

**Note:** 1, Placebo; 2, Dob; 3, Mil; 4, LEV 0.075ug/kg/min;5, LEV 0.1ug/kg/min;6, LEV 0.2ug/kg/min. *P* value > 0.05 indicates that there is no significant inconsistency which means the direct, indirect and network comparison is consistent.

- 1. **Homogeneity hypothesis: Heterogeneity analysis**
     1. **Function used:** *mtc.anohe*
     2. **Results**


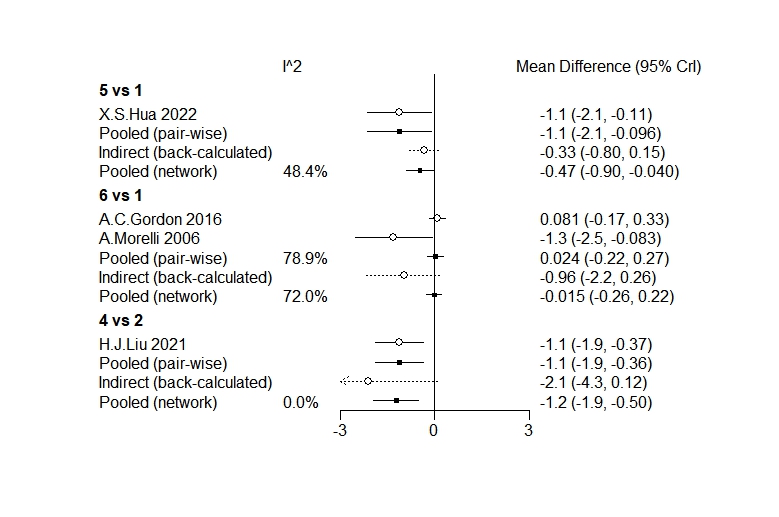


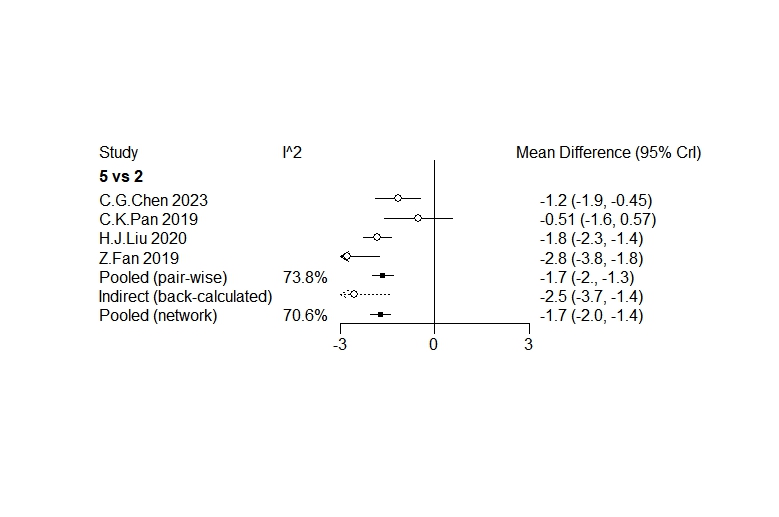


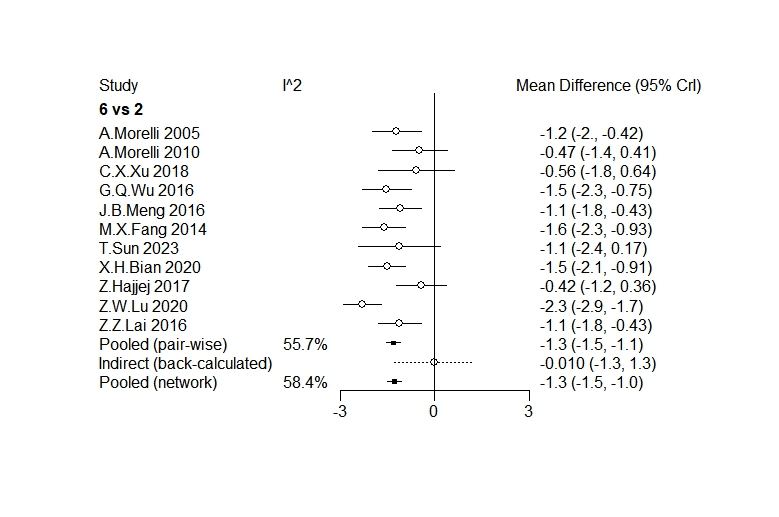


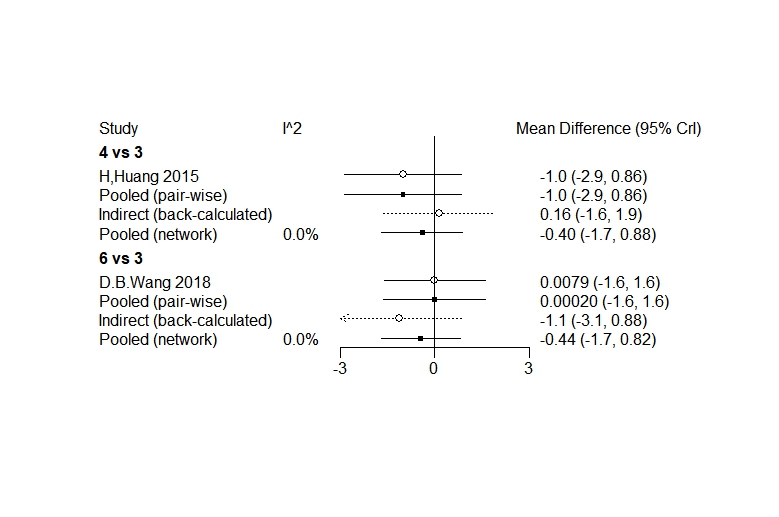


**Figure SII-4.3.2 Heterogeneity analysis of the Bayesian analysis of lactic acid**

**Note:** From the figure we could see, there exists no significant heterogeneity between any two arms, which allows the use of fixed effect model to estimate the pooling results.

**III 28-day mortality**

In this part, we used "gemtc" package(version 1.0-1) in R(version 4.2.3) to conduct the Bayesian Meta analysis.

1. **Building Network**
   1. **Function used:** *mtc.network*
   2. **Results:**


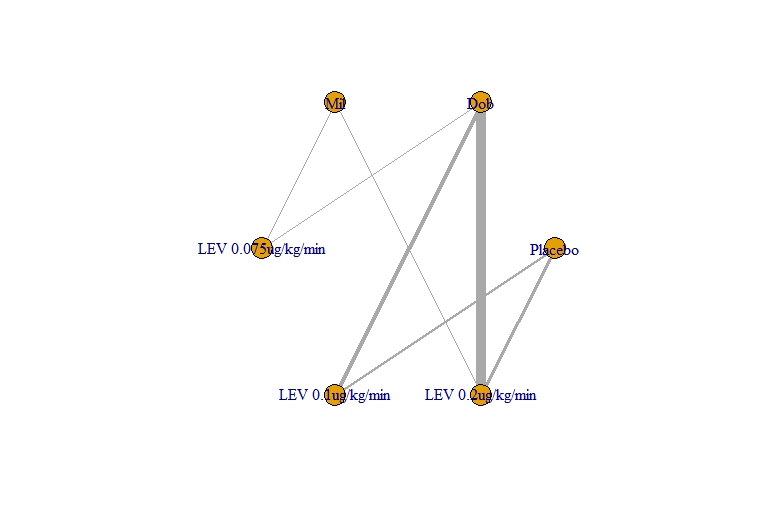


**Figure SIII-1 Network of 28-day mortality**

1. **Modelling and Running**
   1. **Function used:** *mtc.model, mtc.run*
2. **Results Presentation:**
   1. **Forest plot (Placebo was set as the baseline to calculate the relative effect among arms)**


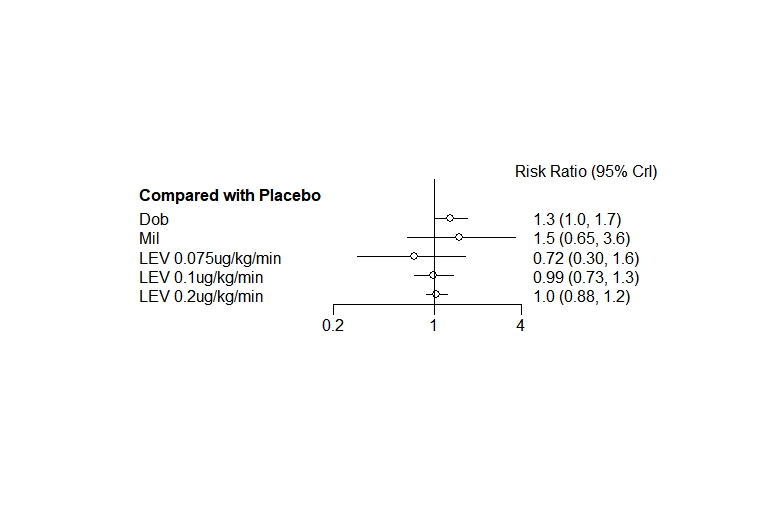


**Figure SIII-3.1 Relative Effect Forest Plot of 28-day mortality (baseline: placebo)**

**Abbreviation:** CrI, credible interval.

- 1. **League table**

**Table SIII-3.2 League Table of 28-day mortality**

| Placebo |  |  |  |  |  |
| --- | --- | --- | --- | --- | --- |
| **0.77 (0.6, 0.99)** | Dob |  |  |  |  |
| 0.67 (0.28, 1.53) | 0.87 (0.36, 1.98) | Mil |  |  |  |
| 1.38 (0.61, 3.38) | 1.78 (0.82, 4.26) | 2.07 (0.77, 6.18) | LEV 0.075ug/kg/min |  |  |
| 1.01 (0.74, 1.37) | **1.31 (1.01, 1.71)** | 1.51 (0.64, 3.73) | 0.73 (0.3, 1.68) | LEV 0.1ug/kg/min |  |
| 0.96 (0.82, 1.13) | **1.25 (1.01, 1.55)** | 1.44 (0.64, 3.42) | 0.7 (0.29, 1.57) | 0.95 (0.71, 1.29) | LEV 0.2ug/kg/min |

- 1. **Rank of probability**


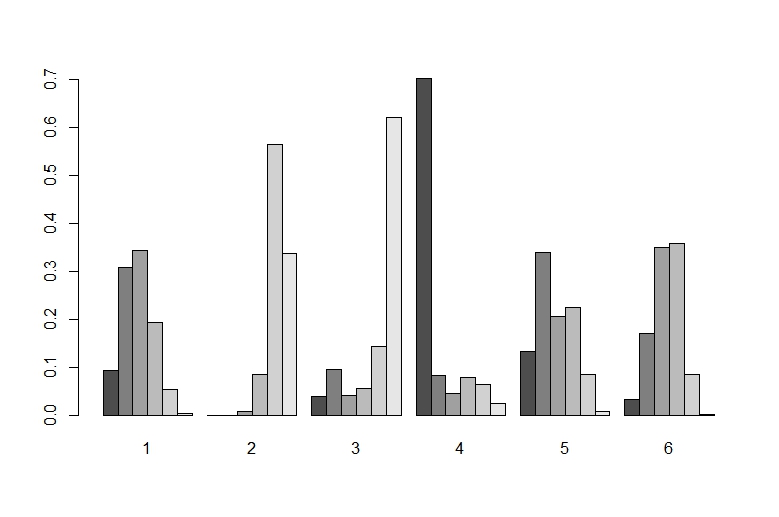


**Figure SIII-3.3 Bar chart of rank of probability of 28-day mortality.**

**Notes:** 1, Placebo; 2, Dob; 3, Mil; 4, LEV 0.075ug/kg/min;5, LEV 0.1ug/kg/min;6, LEV 0.2ug/kg/min.

- 1. **SUCRA rank of probability**

**Table SIII-3.4 SUCRA rank of probability of 28-day mortality.**

| Intervention | SUCRA |
| --- | --- |
| Placebo | 63.57% |
| Dob | 15.43% |
| Mil | 19.31% |
| LEV 0.075ug/kg/min | 84.05% |
| LEV 0.1ug/kg/min | 63.69% |
| LEV 0.2ug/kg/min | 53.94% |

**Abbreviation:** SUCRA, surface area under the cumulative ranking curve.

1. **Quality Control**
   1. **Convergence test: PSRF value**


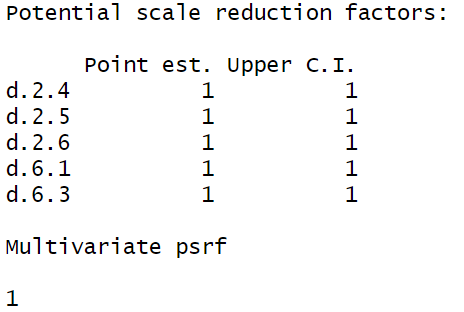


**Figure SIII-4.1 PSRF of the Bayesian analysis of 28-day mortality**

**Abbreviation:** PSRF, Potential scale reduction factors.

**Note:** The closer the PSRF value is to 1, the better. >1.05 indicates unsatisfactory convergence.

- 1. **Consistency hypothesis: inconsistency test.**
     1. **Function used:** *mtc.nodesplit*
     2. **Results:**

**
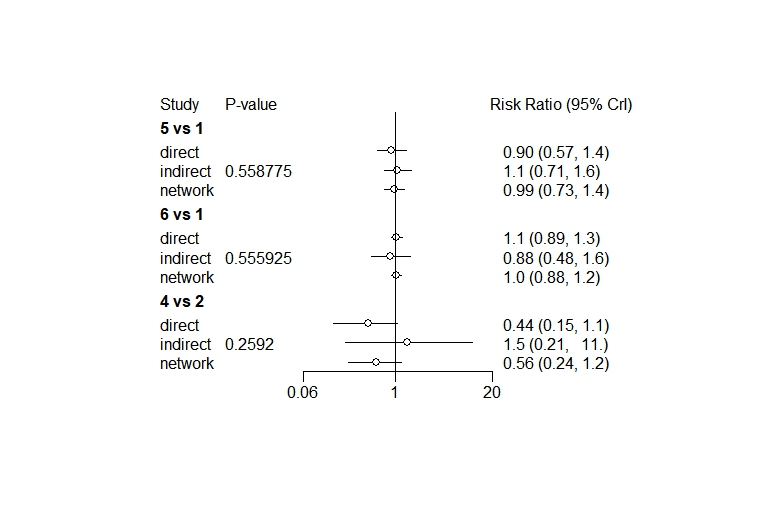
**

**
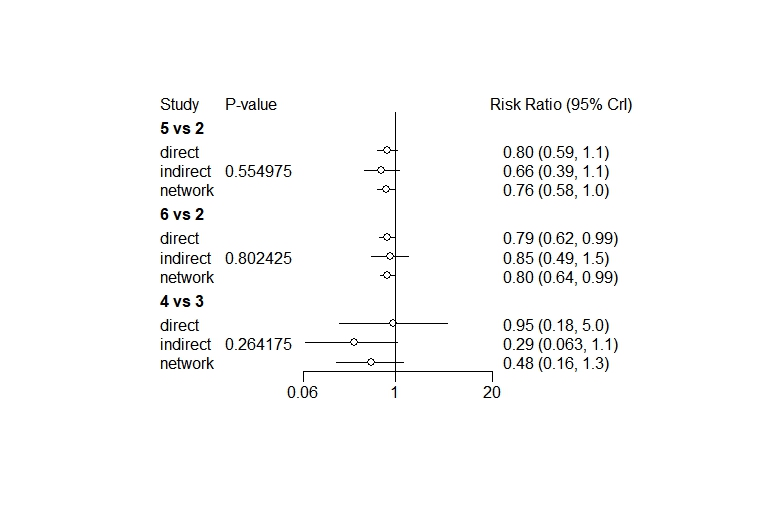
**

**
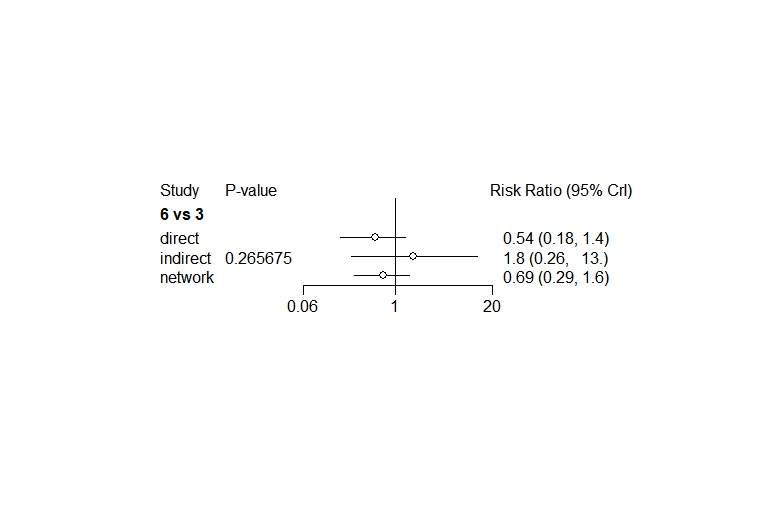
**

**Figure SIII-4.2.2 Inconsistency test of the Bayesian analysis of 28-day mortality**

**Note:** 1, Placebo; 2, Dob; 3, Mil; 4, LEV 0.075ug/kg/min;5, LEV 0.1ug/kg/min;6, LEV 0.2ug/kg/min. *P* value > 0.05 indicates that there is no significant inconsistency which means the direct, indirect and network comparison is consistent.

- 1. **Homogeneity hypothesis: Heterogeneity analysis**
     1. **Function used:** *mtc.anohe*
     2. **Results**


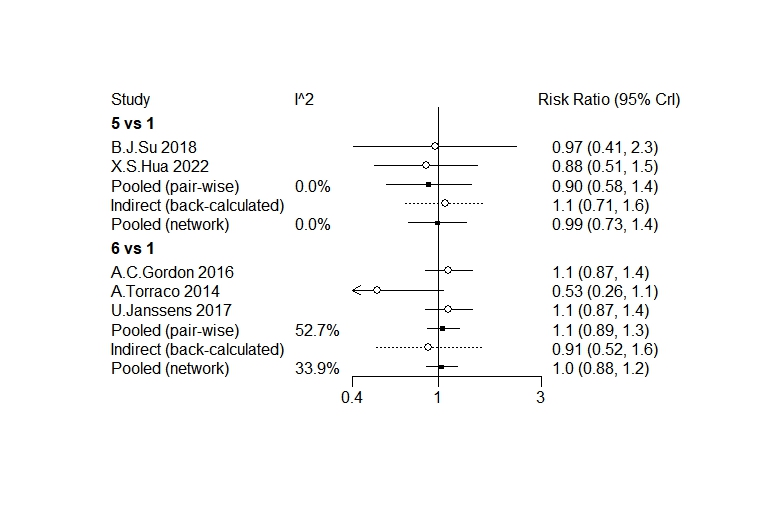


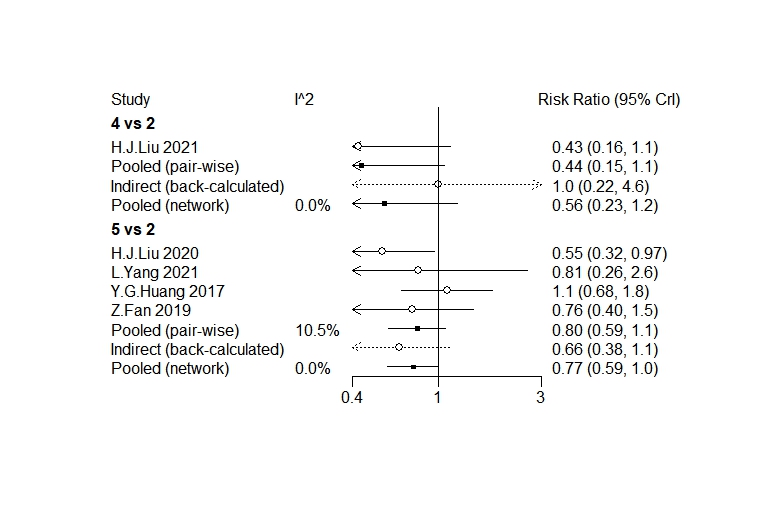


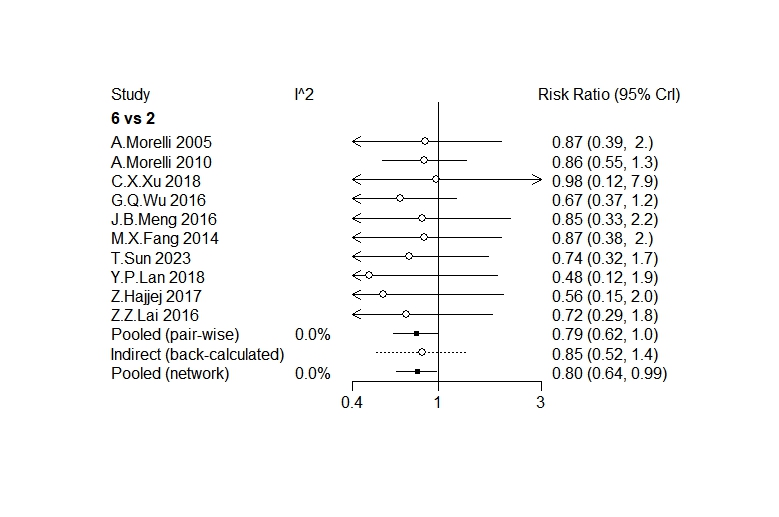


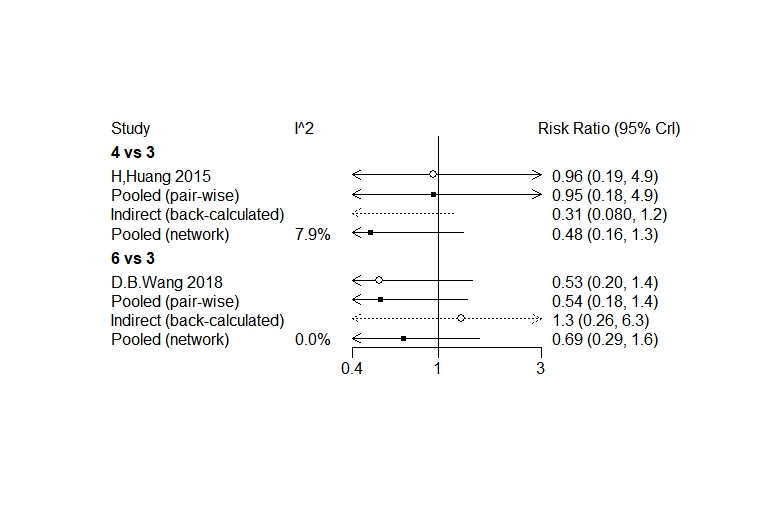


**Figure SIII-4.3.2 Heterogeneity analysis of the Bayesian analysis of 28-day mortality**

**Note:** From the figure we could see, there exists no significant heterogeneity between any two arms, which allows the use of fixed effect model to estimate the pooling results.
